# Editorial

**Published:** 1912-10

**Authors:** 


### EDITORIAL

The Business Office of the *Journal-Record* is 23 West Alabama Street.

The Editorial Office is 1014-15 Atlanta National Bank Building.

Address all Business Communications to *Journal-Record of Medicine*, 23 West Alabama Street.

Make remittances payable to *The Journal-Record of Medicine.*

On matters pertaining to the Editorial and Original Communications, address Edgar G. Ballenger, M. D., Atlanta, Ga.

Reprints of Original Articles will be furnished at cost price. Requests for the same should always be made in THE MANUSCRIPT.

We will present, postpaid, on request to each contributor of an original article, twenty (20) marked copies of *The Journal-Record of Medicine* containing such article.

### RESTRICTED MATERIA MEDICA.

Le Fevre has recently called attenttion to the desirability of a more restricted Materia Medica and nearly all practitioners and teachers of Medicine realize full well the necessity of such restriction. Undoubtedly the medical student would develop into a more competent physician if he were taught thoroughly the uses and methods of application of a few remedies of recognized value, rather than given a large number of drugs, from which, with inexperience, he has to select the supposedly useful ones. Greater skill in diagnosis with the accurate application, a few clearly indicated drugs with a correction of the patient's habits, diet, hygenic surroundings, exercise, bathing, etc, etc, would give better results by far, than our present methods. At the same time it would make the course taken by the medical student less complicated so that he would have a few clearly defined measures instead of being confused by a mass of half digested facts; many of which are of doubtful value.

In reality, the entire course of medicine should be made simpler and more concise and the student should be taught more about the essentials, stripped of complicated theories, until he gets his bearings and a broad, general view. Later the details of each picture may be filled in with a better understanding and therefore a more rational application of therapeutics, especially if the methods of laboratory diagnosis are used as a guide.

